# Draft genome sequence of *Lactococcus lactis* subsp. *lactis* RTIII518: a potent dairy starter isolated from traditional *lait caillé* in Burkina Faso

**DOI:** 10.1128/MRA.00931-23

**Published:** 2023-12-04

**Authors:** Geoffroy Romaric Bayili, Maria Diaz, Charles Parkouda, Hagrétou Sawadogo-Lingani

**Affiliations:** 1 Département Technologie Alimentaire (DTA), Institut de Recherche en Sciences Appliquées et Technologies (IRSAT), Centre National de la Recherche Scientifique et Technologique (CNRST), Ouagadougou, Burkina Faso; 2 Gut Microbes and Health Institute Strategic Programme, Quadram Institute Bioscience, Norwich, United Kingdom; The University of Arizona, Tucson, Arizona, USA

**Keywords:** traditional* lait caillé*, *Lactococcus lactis*, endogenous microorganism, spontaneous fermentation, draft genome sequence

## Abstract

*Lactococcus lactis* subsp. *lactis* RTIII518 was isolated from *lait caillé*, a traditional fermented milk product from Burkina Faso. The draft genome size is 25,554,260 base pairs with 76 contigs, 34.26% average content of GC, 50 tRNAs, 4 rRNA, and 2,498 coding genes.

## ANNOUNCEMENT

*Lactococcus lactis* is selected for use in the food sector based on its fermentative metabolism and ability to produce unique compounds such as lactic acid and aroma compounds (1 – 3).

Lactococcus, from lait caillé fermented milk originating from the rural site of Tolotama (Burkina Faso), was enumerated on M17 agar (Liofilchem, Italy) after aerobic incubation (48 h, 37°C). Then, the strain RTIII518 was picked as a single colony following purification by successive streaking and identified (4) by 16S rRNA gene sequencing (GenBank accession number: MH431827). The strain was found to ferment milk faster than the traditional process with rheological and sensorial properties similar to yogurt (5, 6). The strain was stored in glycerol at −80°C (4). There were approximately four passages between the original freezing stock and genomic DNA extraction.

Genomic DNA was extracted from a pure culture, grown aerobically on M17 agar medium (24 h, 30°C), according to the cetyltrimethylammonium bromide-based extraction protocol (7) and was sequenced at the Earlham Institute (Norwich, UK). Library preparation was done with the Nextera XT DNA library Prep kit (Illumina) and sequencing (150 cycles) was performed with the Illumina NextSeq platform. The library resulted in 5,856,628 reads and 878,494,200 bp. These reads were quality trimmed with BBDuk (version 38.68). Reads with a length below 100 bp or an average quality of less than phred 20 were discarded. *De novo* assembly was performed with SPAdes (version 3.11.1) (8) and annotated using the National Center for Biotechnology Information-Prokaryotic Genome Annotation Pipeline (NCBI-PGAP), version 6.6 (2023) (9) through the NCBI Genome submission portal. The BV-BRC PATRIC web server (version 3.31.12) (10) was also used for complementary annotation (using the RAST tool kit) to create a genome circular map and to find the closest reference strain using the “Similar Genome Finder Service” (11). The presence of bacteriocin genes was investigated with BAGEL4 and antiSMASH (version 5.0) (12, 13). Default parameters were used for all software unless otherwise specified.

**Fig 1:**
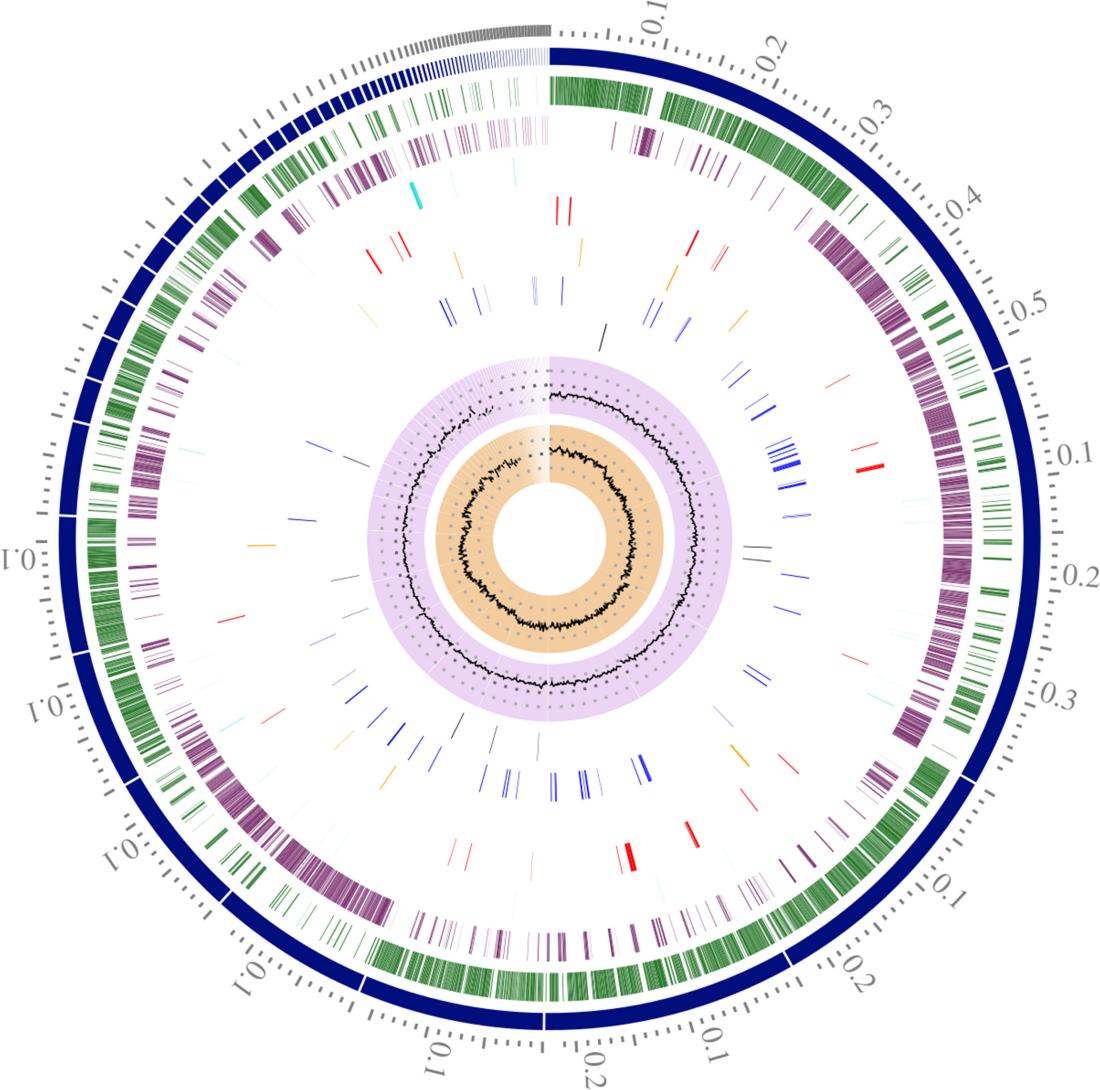
Circular view of the genome of *Lactococcus lactis* subsp. *lactis* RTIII518 strain. Tracks are displayed as concentric rings, from outermost to innermost: 1, reference position in the genome; 2, position and order of the 76 assembled contigs; 3, CDS-forward strands; 4, CDS-reverse strand; 5, non-coding features; 6, anti-microbial resistance genes; 7, virulence factors genes; 8, genes for transporters; 9, drug-targets; 10, GC content; and 11, GC skew.

The PATRIC genome analysis tool indicated a genome size of 2,554,260 bp with an average GC content of 34.26%. The genome was assembled in 76 contigs, with an N_50_ of 226,718 bp. The genomic features are represented in the circular map (Fig. 1). The NCBI-PGAP determined that the genome contains 50 tRNAs and 4 rRNA (two of 5S, one of 16S, and one of 23S), 2,574 total coding sequences (CDSs), 2,498 genes (coding), 4 ncRNAs, and 76 pseudogenes. The closest reference strain (Mash distance: 0.0230043) was *Lactococcus lactis* subsp. *lactis* Il1403 (GenBank accession: AE005176). Potential sites for bacteriocin production were found. This could be of interest for food safety since the strain was suggested for use as a starter.

## Data Availability

This draft genome shotgun project has been deposited at DDBJ/ENA/GenBank under the accession JAVKYZ000000000. The version described in this paper is version JAVKYZ010000000. The NCBI BioProject accession number is PRJNA1014387. The reads data are available in the SRA under the accession number: SRR26171692.
